# Low Light/Darkness as Stressors of Multifactor-Induced Senescence in Rice Plants

**DOI:** 10.3390/ijms22083936

**Published:** 2021-04-11

**Authors:** Ahmed G. Gad, Habiba, Xiangzi Zheng, Ying Miao

**Affiliations:** 1 Fujian Provincial Key Laboratory of Plant Functional Biology, College of Life Sciences, Fujian Agriculture and Forestry University, Fuzhou 350002, China; ahmedgomaagad@yahoo.com (A.G.G.); habiba.geb.ru@outlook.com (H.); 000q816102@fafu.edu.cn (X.Z.); 2 Plant Pathology Department, Faculty of Agriculture, Alexandria University, El-Shatby, Alexandria 21545, Egypt

**Keywords:** leaf senescence, phytohormone, chlorophyll degradation, senescence-associated genes, dark-induced senescence, low light

## Abstract

Leaf senescence, as an integral part of the final development stage for plants, primarily remobilizes nutrients from the sources to the sinks in response to different stressors. The premature senescence of leaves is a critical challenge that causes significant economic losses in terms of crop yields. Although low light causes losses of up to 50% and affects rice yield and quality, its regulatory mechanisms remain poorly elucidated. Darkness-mediated premature leaf senescence is a well-studied stressor. It initiates the expression of senescence-associated genes (SAGs), which have been implicated in chlorophyll breakdown and degradation. The molecular and biochemical regulatory mechanisms of premature leaf senescence show significant levels of redundant biomass in complex pathways. Thus, clarifying the regulatory mechanisms of low-light/dark-induced senescence may be conducive to developing strategies for rice crop improvement. This review describes the recent molecular regulatory mechanisms associated with low-light response and dark-induced senescence (DIS), and their effects on plastid signaling and photosynthesis-mediated processes, chloroplast and protein degradation, as well as hormonal and transcriptional regulation in rice.

## 1. Introduction

For about 65% of the world’s population, rice is a primary food source. Almost 95% of rice in Asia grows as a rainy season crop (i.e., it grows in the wet season when solar radiation decreases to 40–60% of its peak), and is regularly exposed to low light intensity and efficiency at different stages of development [1,2]. The presence of sub-optimal solar radiation during the growing season acts as an abiotic stress, limiting the rice yield to between 40% and 50% [1]. The reduction in solar radiation caused by clouds affects the photosynthesis process [3,4], and this impact may be graded according to the length of short-term and long-term responses. In addition, short-term exposure acclimatization may be differentiated from developmental acclimatization that shows anatomical changes and physiological acclimatization, including changes in protein contents and chloroplast pigments, as reviewed in [5].

Leaves are the primary energy-harvesting site and the major organ for plant development and growth, whose color is an important agricultural trait. Leaf color changes attributable to chlorophyll degradation and disassembly are the first phenomena of dark- and low-light-induced senescence, which are chiefly regulated by pheophorbide a oxygenase (PAO)/phyllobilin pathway [6,7]. The green color of normal plants is correlated with chloroplast pigments, which play a major role in solar energy absorption and photosynthesis. In addition, as they are the food factories of plants, chloroplasts are the primary nutrient site for remobilization to the sinks and newly growing organs during senescence, as they are the main source of leaf nitrogen content, and up to 70% of the leaf nitrogen is sequestered in the chloroplasts [8].

Natural leaf senescence at the end of a plant’s life is an age-dependent process, which comprises programmed disassembly and degradation of the macromolecules that are used for transferring nutrients from senesced leaves to young organs [7]. Metabolites and phytohormones are endogenous factors that tightly regulate plant senescence [9,10,11]. Meanwhile, light, deep shading, drought, high salinity, and pathogens constitute external triggers [12,13,14,15]. With the concerted behavior of many pathways, premature leaf senescence is highly regulated, leading to the initiation of chlorophyll degradation enzyme-encoding gene (CDG) expression, where the enzymes are involved in nutrient remobilization (proteases, nucleases) and related transcription factors (TFs) [16,17]. Moreover, the expression of senescence-associated genes (SAGs) is also initiated [18]. The identification of stay-green mutants is one informative approach to understanding the regulatory mechanisms of leaf senescence. Notably, the stay-green phenotype may lead to an increase in rice yield during the grain-filling stage [19], suggesting the potential of the stay-green trait as a useful tool for boosting crop yield.

Light is an essential regulator of plant growth and development, in addition to being the fundamental energy source. Although both light quantity (fluency) and quality (wavelength) are efficient drivers for plant life, light quality is the leading regulator of plant growth and morphological processing [20]. Research related to the effects of individual light quality on plant growth and development has received considerably more attention. The discovery of the red light (R)-stimulated germination of lettuce seeds [21] was considered as a critical moment in signaling- and production-related research. In addition to conventional technologies, newly developed technologies such as transcriptomics, proteomics, and metabolomics [11,22] have also proven that light modulates various physiological processes and signaling cascades of plants, which thus affects almost all cellular levels. Low light, however, seems to alter both light quantity (fluency) and quality (wavelength), which is a complicated parameter. Recently, it has become pivotal to deeply and comprehensively investigate plant metabolism during low-light conditions, given the possible effect of light quality transitions on rice yield and quality.

This review attempts to elucidate the regulation of low-light- and dark-induced senescence, highlighting new molecular mechanisms. Since the molecular mechanisms underlying dark-induced senescence have been extensively explored, we will concentrate on recent advances explaining low-light/dark-induced regulatory mechanisms. Premature leaf senescence in rice is associated with plastid signaling and photosynthesis-mediated processes, the chloroplast and protein degradation pathways, as well as hormonal and transcriptional regulation.

## 2. Results

### 2.1. Rice Growth and Low Light/Darkness

Cultivated rice (*Oryza sativa* L.) emerged over 10,000 years ago from the middle region of the Pearl River in Southern China [23]. Eventually, it spread throughout the world, becoming one of the major crops along with wheat and maize, which support the global population of around seven billion people. Owing to its good adaptability to the warm and humid monsoon climate of Asia, rice has become the dominant crop in East, Southeast, and South Asia, where the tropical regions practice the development of irrigation systems and the intensive utilization of land, such as double or triple rice cropping. Rice is a sun-loving crop with high light requirements ranging between 30,000 and 50,000 lux depending on the developmental stage. During the tillering and booting stages, its maximum light requirements are 60,000 and 80,000 lux, respectively. Thus, low light can severely affect its vegetative and reproductive growth [24].

The photosynthetically active radiation (PAR) derived from solar irradiance is banded over the 400–700 nm wavelength range, as the spectral absorption of photosynthetic pigments peaks at these specific wavelengths [25]. Deep shading (cloudy) conditions lead to a reduction in total solar radiation by 40,000–50,000 lux [26], which is accompanied by changes in light intensity, duration, and composition [27]. Moreover, sunshine hours are shortened to below 1200 h per year, although rice requires about 1500 bright sunshine hours from transplanting to maturity [28,29,30]. Additionally, the shading condition is characterized by a low red/far-red light (R-FR) ratio (<1), leading to the suppression of R/FR-absorbing phytochrome B (PhyB) [31,32]. Generally, previous reports have shown that deep shading severely limits rice yield by reducing the intensity and quality of light absorbed by leaves [29,32,33]. Given the scarcity of research describing low-light molecular acclimation in rice, it is necessary to spotlight darkness, the closest stressor.

Chlorophyll pigments are the main absorbers and transducers of solar energy. Comparative studies between varieties that are tolerant and sensitive to low light indicated that tolerant varieties exhibited significantly high chlorophyll (a and b) contents and significantly low chlorophyll a/b ratios, respectively. Subsequently, the efficient photosynthetic rate under low-light treatments enhances the potential capacity of antioxidant enzymes. Some evidence in the literature has suggested that plants under light shading mediate some of these processes, such as increasing subunits of ferredoxin, cytochrome P450 and PS1 reaction cores, which are accompanied by increases in some metabolites (particularly carbon metabolites), and thus establish equilibrium under shading conditions that result in a low-light response [34], with no or low substantial impact on yield. Similarly, tolerant rice varieties have been shown to exhibit higher chlorophyll a and b contents and lower chlorophyll a/b ratio than the light-sensitive varieties [6], in addition to maintaining a higher capacity of antioxidants.

### 2.2. Mechanism of Low-Light Response in Rice

There has been an increasing number of studies concerned with how low light affects plant growth and biomass, which range from chloroplast morphology to metabolic pathway and gene expression regulation. Shading treatment not only reduced chloroplast counts by 19%, but also greatly affected the chloroplast morphology. The formation of edema-like structures was noted, which changed the chloroplast shape to nearly circular, accompanied by significant decreases in grana and grana lamella number estimates by 17% and 31%, respectively, at the tasseling stage [35], in addition to a reduction in yield components (kernel, ear numbers) causing a reduction in total yield.

Light differentially orchestrates the gene expression associated with metabolic pathways in rice. For example, blue light initiates the expression of photosynthesis-associated genes [36], whereas dark conditions promote the utilization of stored carbohydrates [37]. The topological analysis of gene expression data with a rice genome-scale metabolic model further revealed that genes related to phytohormones such as abscisic acid, ethylene, gibberellins, and jasmonic acid, are the primary biomarkers of light-mediated regulation, and subsequent analysis of the promoter regions of the associated genes identified several light-specific TFs. Finally, the transcriptional control of rice metabolism by red and blue light signals was assessed by integrating the transcriptome and metabolome data with constraint-based modelling [36].

According to a recent research work, the response of differential molecular sets to low-light stress involves gene expression, and their collective role in maintaining normal metabolic activities can provide evidence for low-light response [6]. The results of this work indicate that genes encoding chlorophyll a/b binding (CAB) proteins, light-regulated protein (LRP), sedoheptulose 1,7-bisphosphatase (SBPase), plant metallothionein 15 (MT15), and transcription factor PCL1 were up-regulated in rice varieties with low-light tolerance (i.e., *Swarnaprabha*). On the contrary, decreased expression of PCL1 was noted in the variety with low-light sensitivity (*IR8*). PSII-PSB27-H1 (a portion of the PSII complex) was found to be down-regulated in *IR8*, which is known to participate in photosynthetic energy metabolism and photo-damaged PSII repair [38]. It also plays a role in promoting the assembly of manganese clusters in PSII [39]. Recently, the OsNAP transcription factor with a NAC-domain-containing protein displayed accelerated leaf senescence at the grain-filling stage, which was expected to be involved in low-light molecular regulation [40].

Proteome analysis showed that the chloroplast and Calvin cycle proteome were significantly affected by low light. The catalytic enzymes of the Calvin cycle pathway were remarkably down-regulated, particularly the glyceraldehyde-3-phosphate dehydrogenase (GAPDH). The over-expression glyceraldehyde-3-phosphate dehydrogenase B subunit (GAPB) increased the chlorophyll content under low-light stress, indicating that GAPB could enhance the tolerance to low-light stress by increasing the chlorophyll accumulation and the photosynthetic rate [41].

Additionally, low light intensity could affect photosynthesis- and energy-related proteins [42,43]. In a study conducted on maize plants, shading conditions decreased the abundance of sucrose synthases and carbohydrate substances (fructose-bisphosphate aldolase, starch synthase, and isoflavone reductase) initially by inhibiting PSI and dark reactions, thus enhancing light energy utilization by the accumulation of electron-transfer-related proteins and alleviating the detrimental effects of shading on plant physiological processes. An investigation of differentially abundant proteins (DAPs) revealed a significant increase in PSII proteins and the inhibition of starch synthesis and CO_2_ fixation, which led to damaged photosynthetic apparatus and consequently reduced biomass and grain yield during late growth stages [34]. Shading has recently been reported to decrease the grain yield of fragrant rice, which has been alleviated by the exogenous application of γ-amino butyric acid (GABA) to non-protein amino acids [44]. As a signaling factor, GABA is involved in many abiotic stress responses [45], which is expected to alleviate the shade-stress effect on fragrant rice through modulation of the antioxidant system (SOD and CAT).

### 2.3. Mechanisms of Dark Response in Rice

Dark-induced premature leaf senescence is a well-studied stressor. It initiates the expression of SAGs that are involved in chlorophyll breakdown and degradation. The molecular and biochemical regulatory mechanisms of premature leaf senescence show significant levels of redundant biomass in the complex pathways. Darkness is a severe form of light stress that causes leaf yellowing, chlorophyll degradation, changes in hormonal dynamics, cellular structure damage, and altered gene regulation in rice.

#### 2.3.1. Hormonal Dynamics during Dark-Induced Senescence (DIS)

Plant hormones such as auxin, cytokinins, jasmonic acid (JA), abscisic acid (ABA), ethylene, gibberellins, and brassinosteroids are signaling molecules that participate in multiple cellular and physiological processes. In particular, cytokinins, JA, and ABA play crucial roles in DIS regulation. JA and its derivatives were reported to have significant roles in the defense and development regulation processes of plants [46,47,48]. Besides, JA signaling, as an inducible pathway, was found to exert an important function in leaf senescence regulation by targeting some SAGs in *Arabidopsis* [49,50]. Rice jasmonate ZIM-domain protein (OsJAZ8)—a repressor of JA signaling, degradation, and ubiquitination—enables the release and regulation of downstream TFs (e.g., MYC2 and its homologs MYC3/4), which are positively regulated by ANAC019, ANAC055, and ANAC072 TFs in JA-mediated leaf senescence under DIS conditions [51]. After 3 days of dark treatment, the expressions of JA signaling-related genes (*OsMYC2* and *OsCOI1a*) and JA biosynthesis-related genes (*OsLOX2* and *OsAOS1*) were down-regulated in the ethylene-responsive factor (*oserf101*) mutant as compared to WT [52]. Meanwhile, low endogenous JA levels and low expressions of JA-biosynthesis-related genes (*OsLOX2*, *OsLOX8*, *OsHI-LOX*, *OsAOS1,* and *OsAOS2*) were reported in the *osdof24-D* (gain-of-function lines), suggesting a delayed senescing phenotype during DIS [53].

In regards to delayed senescence, the potential functions of cytokinins in growth regulation and senescence suppression have been reported [54,55]. Cytokinin oxidase/dehydrogenase (CKX) is a key enzyme that catalyzes cytokinin degradation. Knock-out mutant (*osckx*) reversely promoted the cytokinins and ABA levels, and exhibited a significant increase in cytokinins levels as compared to WT during dark treatment. On the contrary, decreases in ABA levels were noted, accompanied by down-regulation of ABA-biosynthesizing genes and up-regulation of ABA-degrading genes in the *osckx11* mutant as compared to the WT [56]. Similarly, the expressions of ABA-signaling and biosynthesizing genes were significantly down-regulated in *osmyb102-D* and up-regulated in rice myeloblastosis 102 (OsMYB102)-overexpressing lines, which were accompanied by the activation of abscisic acid 8′-hydroxylase 2-like (ABA8OX2 ), which functions in the ABA catabolic pathway. In contrast, auxin-biosynthesizing and signaling genes were significantly down-regulated [57].

#### 2.3.2. Signaling Molecule Regulation during DIS

Premature leaf senescence and DIS initiate dramatic metabolic changes that are represented by macromolecular hydrolysis and reactive oxygen species (ROS) accumulation [58,59]. Previous studies have declared that sugar levels could act as signaling molecules that control a variety of developmental and senescent processes [37,60,61]. Recently, it has been reported that the exogenous application of sucrose and glucose delayed DIS, accompanied by decreased levels of hexokinase 1 (HXK1) and hexokinase 2 (HXK2), which were involved in hexose-mediated signaling [62].

The initiation of ROS could damage the cellular membranes, causing the rapid degradation of nucleic acids and proteins, subsequently resulting in premature leaf senescence syndrome [63,64]. Timely/periodical scavenging of ROS in an appropriate way is crucial to alleviating oxidative stress [65,66]. Melatonin has been found to play a significant role in balancing ROS. Premature leaf senescence 3 (*pls3*) mutants, which disturb the genes encoding O-methyltransferases that act at the last stage of melatonin biosynthesis, have been reported to show a rapid senescence phenotype during DIS, accompanied by low melatonin levels and subsequently significant ROS accumulation levels as compared to WT. However, the exogenous application of melatonin delayed the senescence in the whole dark-treated plants [67]. In ryegrass (*Lolium perenne*), the exogenous melatonin application was found to phenotypically suppress DIS, which agreed with significantly decreased O_2_ and H_2_O_2_ production levels, as well as reduced malondialdehyde (MDA) and non-enzymatic antioxidant levels. On the other hand, melatonin treatment enhanced the enzymatic activities of CAT and SOD [68].

Proline, an important metabolic signaling molecule, was accumulated in rice leaves during DIS [69,70]. Recently, its role in *Arabidopsis* was investigated, which found that its oxidation could provide energy to compensate for the loss of chloroplasts during DIS until the completion of the remobilization process [71].

#### 2.3.3. Transcriptional Regulation during DIS

Transcriptome analysis of rice leaves after dark treatment revealed that 30% of genes altered their expressions under DIS, including various transcriptionally regulated TF families such as EINs, PIFs, NACs, and MYC/MYBs [72]. Ethylene insensitive (EIN3) is the core component of the ethylene signaling pathway and the positive regulator of senescence in *Arabidopsis* [73,74], and its content is controlled by F-BOX1 and F-BOX2 [75]. It was found that EIN3 accumulation directly activated the expression of SAG29/SWEET15, a sugar transporter, whose over-expression led to defects in pollen tube attraction [76]. OsEIL1, the homolog of EIN3, up-regulated the downstream target gene β subunit of polygalacturonase subfamily (*OsBURP16*) expression by a 270-fold response to dark treatment, causing decreased pectin content and thus affected the cell integrity [77,78]. Regarding another ethylene response factor ERF101, its loss-of-function mutants (*oserf101*) retained their leaves’ green color, which exhibited down-regulation of chlorophyll degrading enzymes (NYC1 and NYC3) and SGR, in addition to NAM and CUC TF candidates, as compared to the WT leaves [52].

Rice NAC TFs with 149 homologous genes [79,80,81] were found to tightly and positively regulate the SAG expression levels under DIS. Loss of function of OsNAC2, OsNAC054, and OsNAC096 exhibited the stay-green phenotype in comparison to the WT. OsNAC2 was found to directly regulate SGR and NYC3; OsNAC054 initiated ABI5 and NYC1, while OsNAC096 increased the ABI5 and EEL expressions. In contrast, the over-expression lines accelerated the leaf yellowing under DIS [82,83].

**Figure 1:**
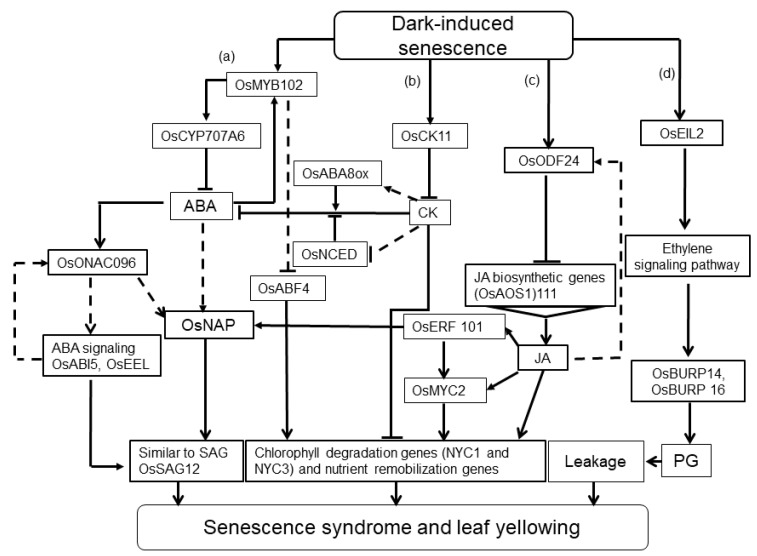
Proposed model for the multilayered regulation of dark-induced senescence (DIS) and possible signaling-mediated pathways: DIS could be redundancy-regulated within overlapped pathways, and differentially modulated within ABA pathways as follows: OsONAC096 positively regulates DIS by up-regulating the ABA signaling expression and thus initiates senescence-associated genes (SAGs); on the contrary, OsMYB102 negatively modulates DIS during its function; OsCYP707A6 disrupts ABA levels and downstream transcription factors (TFs) such as OsNAP and OsONAC096, or directly inhibits OsABF4, which directly targets SAGs and NYC1 (**a**). OsCKX11 activates irreversible cytokinin degradation. Mutation of OsCKX11 decreases ABA accumulation, probably by down-regulating the expression of OsNCED (ABA biosynthetic gene), and up-regulating the expression of *OsABA8ox* (ABA catabolic gene). Accumulated cytokinin and depressed ABA levels lead to the inhibition of chlorophyll degradation and maintenance of photosynthesis (**b**). Similarly, JA could modulate DIS and initiate the expression of *OsERF101*, which up-regulates the *OsMYC2* expression and consequently initiates chlorophyll-degrading genes or is cross-talked with OsNAP, a key ABA pathway TF, and thus regulates the SAG expression. On the contrary, OsDOF24 negatively regulates DIS by inhibiting the expression of JA biosynthetic gene *allene-oxide synthase1* (*OsAOS1*) and results in JA level disruption, which could be an inducer for some other TFs such as OsERF101 and OsMYC2 (**c**). The ethylene-signaling pathway may also mediate DIS regulation during the up-regulation of *OsEIL2*, which functions upstream of OsBURP14 and OsBURP16, the members of the β subunit of polygalacturonase subfamilies, and thus reduces the pectin content by enhancing polygalacturonase (PG) activity (**d**).

The gain-of-function lines of DOF TFs, as a negative regulator of both age and DIS, exhibited higher chlorophyll content and decreased levels of CDGs (*NYC1*, *NYC3* and *SGR*), indicating the inhibition of JA biosynthetic genes attributed to their binding to the *OsAOS1* promoter [53] (Figure 1). DOF TFs were also involved in various stress responses [84,85,86,87].

The myeloblastosis (MYB) TF family was found to implement the regulation of various plant-related physiological processes, including biotic and abiotic stress responses, through phytohormones, as well as metabolism signaling, cell cycle regulation [88,89,90], and senescence regulation. *Atmyb44* knock-out mutants accelerated leaf yellowing during senescence [91]. Constitutive expression of *OsMYB102* exhibited a delayed senescence phenotype against dark treatment and retained the green color through the regulation of the ABA signaling pathway, which activated the expression of an ABA catabolic gene (*OsCYP707A6*) and CDGs (*OsNOL, OsNYC1, OsNYC3,* and *OsRCCR1*) [57].

The S40 family, DUF584-domain-containing proteins, was reported to modulate senescence in both *Arabidopsis* and barley [92,93]. In *Arabidopsis*, *AtS40-3* expression was induced during dark treatment, while the T-DNA insertion mutant (*ats40-3a*) showed a stay-green phenotype [92]. The barley HvS40 was first identified as a SAG due to its elevated mRNA level during DIS of detached leaves. This family has 16 homologous members in rice [94]. The 4 candidates of these 16 genes, namely *OsS40–1*, *OsS40–2*, *OsS40–12*, and *OsS40–14*, were up-regulated in response to dark treatment, showing a clear yellowing phenotype as compared to the control [95].

#### 2.3.4. Protein Degradation during DIS

DIS is a typical phenotype characterized by yellowing and chlorophyll degradation. Transcriptome analysis of dark-treated rice seedlings showed down-regulation of key chlorophyll synthetases and leaf color-regulating genes [72]. For instance, *OsChlI* and *OsChlH* were reported to encode the CHLI and CHLH subunits of Mg^2+^-protoporphyrin IX chelatase (Mg^2+^-chelatase) [96,97], while *OsYGL1* encodes the chlorophyll synthase [98]. Their protein levels were decreased in the DIS of detached leaves, which was accompanied by the enhanced activities of acid proteinase, carboxypeptidase, and aminopeptidase [99]. Chlorophyll and chloroplast protein breakdown is considered a massive process in senescing leaves.

Chlorophyllases and proteases are the key enzymes in chlorophyll catabolism. The majority of senescence-associated proteases have been characterized as serine, cysteine (Cys), aspartic, and metalloproteases [8,100]. Additionally, the up-regulation of vacuolar processing enzyme (VPE) genes was detected during senescence [101]. Elevated levels and activities of serine, cysteine, aspartic, and metalloproteases were detected in senescing leaves [102]. *SGR* is a senescence-inducible gene that encodes a chloroplast protein with conserved amino acid sequences. It was elucidated that SGR has a specific affinity to LHCPI and LHCPII. The *sgr* mutant losts the ability to destabilize SGR–LHCPII complexes, which is required for the disassembly of chlorophylls (Chls) and light-harvesting complex proteins (LHCPs) during DIS. Taken collectively, SGR initiates the degradation of Chl and LHCP complexes via proteases and Chl catabolic enzymes [103].

Non-yellow coloring 1 (*NYC1*) and its homolog NYC1-like (*NOL*) genes encode Chlb reductase, which plays a role in the conversion of Chlb to Chla and thus activates the subsequent Chl degradation pathway. Loss-of-function mutants *nyc1* and *nol* exhibited the stay-green phenotype since the inhibition of Chlb degradation was associated with the stability of light-harvesting complexes and the subsequent retention of well-developed thylakoid, even at late senescence [104]. *NYC3* encodes a plastid-localizing a/b hydrolase-fold family protein with an esterase/lipase motif, whose loss-of-function mutant (*nyc3*) has a more similar phenotype to *sgr* than to *nyc* and *nol* [105]. NYC4, which encodes an orthologous thylakoid formation 1 (THF1) protein in *Arabidopsis thaliana* [106], was involved in Chls and Chl–protein complexes that dismantle the regulation process during DIS. In the loss-of-function mutant (*nyc4*), both the high F_v_/F_m_ value and the stability of PSII core subunits were retained during senescence [107].

Rubisco, the most abundant chloroplast stromal protein, was found to be degraded prior to the dismantling of the chloroplast and thylakoids [108], which could occur outside the plastids in spherical bodies containing Rubisco (i.e., “Rubisco-containing bodies”, RCBs) in senescing leaves of wheat and *Arabidopsis* [109,110]. Cysteine proteases were reported as representative of many senescence-associated proteases [111,112,113]. Cysteine over-expression effectively inhibited the cysteine protease activity in rice and delayed the loss of Rubisco and two Rubisco activases in tobacco [114]. Additionally, antisense experiments demonstrated that CND41, a DNA-binding aspartic protease, has an important role in the breakdown of Rubisco [115].

According to an extant report, leaf senescence and Chl degradation could be regulated via an autophagy-like process, as the rapid leaf senescence1 (*rsl1*) mutant with nucleotide binding site domain-containing protein (NB-ARC/NOD) showed revolutionary conservation of animal death effector cells [116]. This mutant was employed to explore the involvement of programmed cell death (PCD) in plant senescence regulation upon exposure to darkness. The mutant displayed rapid Chl loss (54%) as compared to WT (18%), implying a distinguished phenomenon accompanied by leaf yellowing and Chl collapse. Additionally, transmission electron microscopy revealed the formation of double-membrane vesicles containing chloroplast material, which subsequently fused to vacuoles and degraded in a process resembling RCB formation [63].

Xanthine dehydrogenase (XDH) is the key enzyme in the conversion of xanthine and hypoxanthine to uredines, and is also an effective factor in ROS scavenging [117]. Its activity was affected by various abiotic stresses that initiate senescence [118]. According to a recent report, its involvement in DIS regulation as *xdh3* and *xdh4* mutants exhibited an early senescence phenotype, accompanied by significant increases in O^2−^ and MDA contents [119].

## 3. Conclusions and Prospects

Light is the fundamental energy source and a key regulator of various plant physiological processes, as well as their development and responses to different environmental stimuli. Low light/darkness causes premature leaf senescence of rice, which is globally orchestrated in a multilayered manner, thereby affecting agronomic traits of rice [120]. Recent research stated that low-light-tolerant genotypes could maintain better agronomic and physiological traits [32]. On one hand, various TF gene families have been demonstrated to be involved in Chl and protein degradation in DIS, as well as hormonal dynamics at the transcriptional level. On the other hand, at the time of writing this review, there were no reports describing the low-light-induced senescence at the molecular regulatory level, except comparative transcriptomic research between rice varieties that are sensitive and tolerant to low light [6]. Hence, increased research efforts are needed to unravel how the exact mechanisms and enriched pathways are involved in the regulation of low-light stresses.

Comparison with darkness—the most severe light-related stressor—revealed that there may be overlapping molecular mechanisms between low-light- and dark-induced senescence. We speculate that at the genetic level, the stay-green mutants can be collected and planted in the regions suffering from low light and tested for their agronomic responses. At the cytological and molecular levels, chloroplast structures and their protein homeostasis, as well as retrograde signal transduction, may be more concentrated under low-light conditions. At the biochemical level, hormonal networks and metabolic pathways are involved under low-light conditions.
